# Heat Shock Proteins: Agents of Cancer Development and Therapeutic Targets in Anti-Cancer Therapy

**DOI:** 10.3390/cells9010060

**Published:** 2019-12-24

**Authors:** Chul Won Yun, Hyung Joo Kim, Ji Ho Lim, Sang Hun Lee

**Affiliations:** 1 Medical Science Research Institute, Soonchunhyang University Seoul Hospital, Seoul 04401, Korea; skydbs113@naver.com (C.W.Y.); hyungjoothomaskim@gmail.com (H.J.K.); wlenfl1@naver.com (J.H.L.); 2 Department of Biochemistry, Soonchunhyang University College of Medicine, Cheonan 31538, Korea

**Keywords:** heat shock proteins, cancer therapy, chemoresistance, radiotherapy, immunotherapy

## Abstract

Heat shock proteins (HSPs) constitute a large family of molecular chaperones classified by their molecular weights, and they include HSP27, HSP40, HSP60, HSP70, and HSP90. HSPs function in diverse physiological and protective processes to assist in maintaining cellular homeostasis. In particular, HSPs participate in protein folding and maturation processes under diverse stressors such as heat shock, hypoxia, and degradation. Notably, HSPs also play essential roles across cancers as they are implicated in a variety of cancer-related activities such as cell proliferation, metastasis, and anti-cancer drug resistance. In this review, we comprehensively discuss the functions of HSPs in association with cancer initiation, progression, and metastasis and anti-cancer therapy resistance. Moreover, the potential utilization of HSPs to enhance the effects of chemo-, radio-, and immunotherapy is explored. Taken together, HSPs have multiple clinical usages as biomarkers for cancer diagnosis and prognosis as well as the potential therapeutic targets for anti-cancer treatment.

## 1. Introduction

Cancer is a major public health concern in the world. The report by World Health Organization estimated for 18.1 million new cancer cases and 9.6 million cancer deaths in 2018 [1]. During past few decades, surgical therapy, chemotherapy, radiotherapy, and immunotherapy have considerably been developed. However, ongoing treatments are met with the limitations due to treatment-induced cellular genetic and biochemical changes that confer treatment resistance [2]. Therefore, there is a growing need for developing new therapeutic strategies and discovering molecular targets for effective cancer treatment.

Many studies on cancer biology have revealed lots of potential targets for cancer therapy. One of these is a molecular chaperone, which is a class of proteins known as heat shock proteins (HSPs). HSPs are highly conserved in all mammalian cells and participate in protein quality control by promoting accurate folding of newly synthesized proteins and refolding of denatured proteins under a variety of intracellular and extracellular stressor conditions. Such conditions include sudden changes in temperature, exposure to high levels of reactive oxygen species (ROS), and significant cellular damage affecting structure and stability of proteins [2]. Therefore, HSPs function as the first line of defense against stress-associated cellular challenges. Intriguingly, studies have shown the abnormal expression levels of HSPs in different types of cancer, including prostate, bladder, breast, ovarian, colorectal, and lung cancers [3,4,5]. Since cancer cells require many metabolic needs for progression and invasion, the modulation of HSPs to meet these ends is a requirement [6,7]. Accordingly, the mechanisms by which HSPs regulate cancer cell proliferation, invasion, metastasis, and evasion of apoptosis have been investigated [5,8,9]. HSPs have also been found to promote resistance to anti-cancer therapies such as chemotherapy and radiotherapy [10,11]. Due to these vast associations with cancer development and treatment, targeting HSPs has been suggested as a potential strategy for anti-cancer therapy.

In this review, we comprehensively summarize the cancer-related biological functions of HSPs, particularly focusing on recent findings. In addition, the utilization of HSPs in the context of cancer treatment is discussed as a promising method for effective cancer treatment.

## 2. Overview of Heat Shock Proteins (HSPs) as Agents of Cancer Development

**Figure 1:**
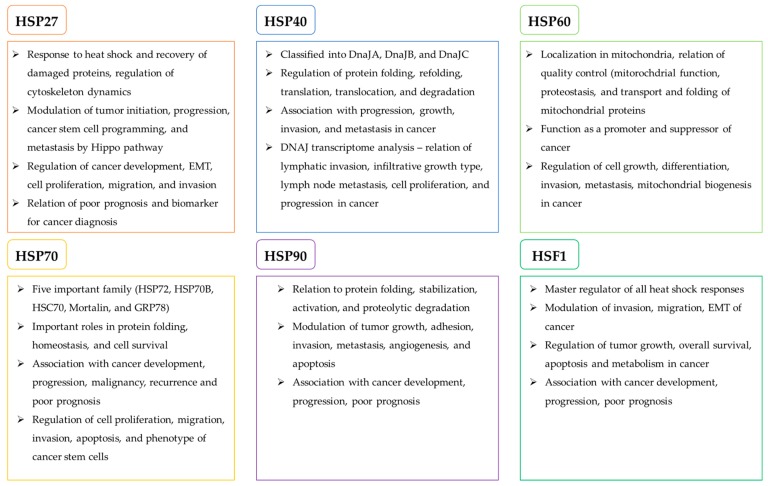
A schema illustrating the overview of heat shock proteins (HSPs) in cancer development.

**Figure 2:**
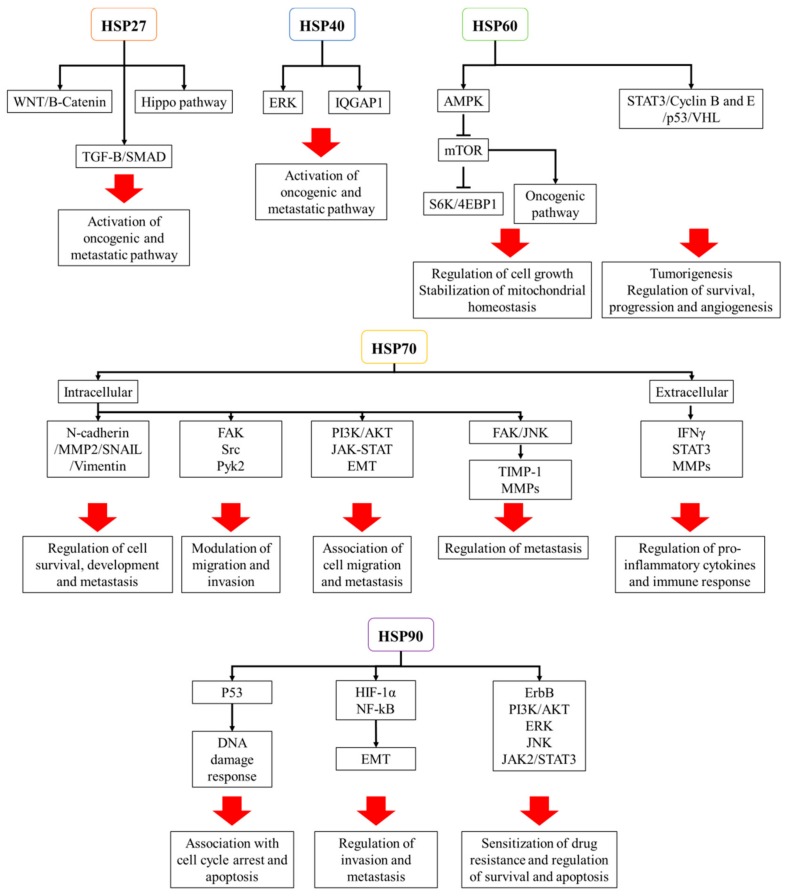
A schema illustrating the multiple signal pathways that are altered by heat shock proteins (HSPs) in cancer.

Heat Shock Proteins (HSPs) are a group of proteins that function to maintain cellular homeostasis in response to stressors such as hypoxia, anoxia, high temperature, drugs, and other chemical agents that induce protein denaturation [3,12]. They facilitate protein folding and maintain protein structures that regulate cellular metabolisms that are essential for cell survival and proliferation. At the same time, cancer cells hijack the protective roles of HSPs during carcinogenesis [13]. HSPs are classified on the basis of molecular weights. In this review, we focus on the best studied HSPs, which are HSP27, HSP40, HSP60, HSP70, and HSP90, and their roles in relation to cancer are summarized in Figure 1 and Figure 2.

### 2.1. Role of HSP27 as an Upstream Regulator of Oncogenic Pathways

HSP27 (*HSPB1*) is a type of small HSPs (12–43 kDa) that work independently of ATP. Induced by heat shock, environmental, and pathophysiological stressors, HSP27 forms multimeric complexes and stabilize denatured or aggregated proteins and return them to their original form [14]. Other functions of HSP27 include direct interference with apoptotic pathway and regulation of cytoskeleton dynamics [15,16]. Whereas its primary function is to promote cellular homeostasis under stressor conditions, overexpression of HSP27 is closely related to tumorigenesis, metastasis, and invasiveness in various cancers such as head and neck squamous cell carcinoma, pediatric acute myeloid leukemia, breast cancer, and colorectal cancer [17,18,19,20]. HSP27 has been identified as an important regulator of the Salvador–Warts–Hippo pathway (Hippo pathway), which controls tumor initiation, progression, cancer stem cell programming, and metastasis. The elevated expression of HSP27 increases the nuclear localization of the Hippo pathway transcription factor, YAP, which activates oncogenic and metastatic pathways, including TGF-B/SMAD, WNT/B-Catenin, and ILK signaling pathways. The Hippo pathway-associated role of HSP27 has been demonstrated in various tumors, including prostate, breast, and lung cancers [21]. Sumoylation, a reversible post-translational modification by the small ubiquitin-related modifier (SUMO) plays an essential role in cancer development through the modulation of DNA damage response, cell cycle progression, metastasis, and apoptosis, accompanied by upregulation of HSP27 [22]. The increased expression of HSP27 also stimulates epidermal growth factor (EGF)-induced cell migration, invasion, and matrix metalloproteinase (MMP) activity as well as the expression of epithelial to mesenchymal transition (EMT) markers via activation or overexpression of AKT, GSK3β, and β-catenin [23]. Furthermore, HSP27-mediated modulation of intracellular calcium influx enhances colorectal cancer cell proliferation, migration, and invasion [24].

### 2.2. Oncogenic Role of HSP40 in Proliferation and Metastasis of Cancer

HSP40 belongs to *DNAJ* family subcategorized into three subclasses, which are DnaJA (*DNAJA*), DnaJB (*DNAJB*), and DnaJC (*DNAJC*) [6]. HSP40 assists in protein folding, unfolding, translation, translocation, and degradation [11,25,26], as well as ATPase activity of HSP70 [27]. Many of the HSP40 family members are overexpressed in numerous human cancer types, such as colorectal, gastric, and lung cancers [28,29,30,31]. Clinicopathologic analyses have shown the markedly increased expression of DnaJA1 in colorectal cancer (CRC) tissues, particularly those of which had developed metastases in lymph node and distant organs. Investigating the functional mechanism of HSP40 in cancer, Yang et al. demonstrated that Hsp40 DNAJ member A1 (DnaJA1) is transcribed by E2F transcription factor 1 and promotes cell cycle progression by inhibiting ubiquitin degradation of cell division cycle protein 45 (CDC45) in CRC [32]. DNAJ member B6 (DnaJB6) has also been elucidated as a poor prognostic factor for CRC patients, where its overexpression was observed in 39% of the CRC patients, especially in those at the stage of cancer IV compared to the stages I–III. In addition, Zhang et al. showed that the mechanism of DnaJB6-mediated enhancement of invasion and metastasis of CRC is by hyper-activating pERK-IQ-domain GTPase-activating protein 1 (IQGAP1) signaling axis. Inhibiting DnaJB6 decreases the IQGAP1 expression and the phosphorylation of ERK in CRC cells in vitro and suppresses the lung metastases of CRC in vivo [29]. In addition, DNAJ member c12 (DnaJC12) is associated with the aggressive phenotype of gastric cancer. The transcriptome analysis has identified that the increased expression of DnaJC12 is correlated with lymphatic invasion, infiltrative growth type, lymph node metastasis, and progression of gastric cancer. The patients with increased levels of DnaJC12, therefore, have higher morbidity and mortality rates, suggesting DnaJC12 as a potent therapeutic target [30].

### 2.3. Role of HSP60 in Cancer Development through Regulation of Mitochondrial Biogenesis

As one of the most conserved proteins from bacteria to mammals, HSP60 (*HSPD1*) plays an essential role in mitochondrial protein import and quality control machinery [11,33]. At the same time, HSP60 may function as a promoter and suppressor of cancer formation depending on disease type. In ovarian cancer (OC), the increased expression of HSP60 enhances tumor progression by stabilizing mitochondrial homeostasis and activating mTOR signaling pathway [34]. In glioblastoma, inhibition of HSP60 leads to the increased formation of reactive species (ROS) in mitochondria, subsequently exerting the complex I inhibitor retenone-induced AMPK activation, which in turn suppresses mTORC1-mediated phosphorylation of S6K and 4EBP1 and deactivates the protein translation machinery and cancer cell growth [33]. The HSP60 family also includes a type II hetero-oligomeric chaperonin (TRiC/CCT), which assists in the folding of about 10% of cytosolic proteins that are not folded by other simpler chaperone systems [35]. TRiC/CCT is associated with pathogenesis of many types of cancers through modulation of TRiC client proteins such as STAT3, cyclins B and E, p53, and Von Hippel-Lindau [36,37,38,39]. TRiC/CCT regulates the folding and function of STAT3 while activation of STAT3 is a common basal property of several solid and hematologic tumors. Abnormal activation of STAT3 induces many oncogenic transcriptional processes related to promotion of cell survival, progression, and angiogenesis [36,40,41,42].

In contrast, the excess formation of ROS that results from mitochondrial dysfunction may also drive primary cancer cells to undergo EMT and enhance their metastatic potentials. When the clinicopathological characteristics of hepatocellular carcinoma (HCC) were analyzed, the expression of HSP60 was significantly decreased in HCC tissues compared to peritumor tissues in the patients with poor prognosis. The low HSP60 cancer/pericancer (C/P) expression ratio was found to be correlated with the dedifferentiation of cancer cell for EMT and malignance. Increasing HSP60 expression, therefore, limits the dedifferentiation process and the metastatic potential of HCC in vitro and in vivo [43]. Similarly, the low HSP60 C/P ratio has also been observed in clear cell renal cell carcinoma (ccRCC) patients. Consistently, overexpressing HSP60 in ccRCC cells restored the mitochondrial function and ROS levels, limiting the metastatic ability of ccRCC in a mouse model [44].

### 2.4. Role of HSP70 in Cancer Development

Encoded by *HSPA* genes, HSP70 consists of 13 members that play essential roles in protein folding, protein homeostasis, and promotion of cell survival under various stresses [45]. In cancer cells, HSP70 functions to induce mitotic signals and suppress apoptosis as well as oncogene induced senescence [46]. The increased expression of HSP70 has been indicated as a poor prognostic marker for a variety of cancers, including breast, lung, ovarian, colorectal, and pancreatic cancers and glioblastoma [45,47,48,49,50]. Among HSP70 family, five members have been especially well examined in association with cancer, which are stress-inducible HSP70s, HSP72 (*HSPA1*) and HSP70B (*HSPA6*), and constitutively expressed HSP70s, HSC70 (*HSPA8*), GRP75/Mortalin (*HSPA9*), and GRP78 (*HSPA5*) [51,52]. Recently, it has been found that HSP72 (HSP70) plays an essential role in organizing kinetochore-associated microtubules for amplified centrosomes, a cancer specific phenotype which, if not stabilized, triggers mitotic catastrophe and apoptosis [53]. In addition, increased levels of HSP70B (HSP70) contribute to breast cancer metastasis through upregulation of mesenchymal markers such as N-cadherin, MMP2, SNAIL, and vimentin [54]. Furthermore, HSC70 overexpression enhances the glioma cell proliferation, migration, and invasion through phosphorylation and activation of FAK, Src, and Pyk2. [55]. As extensively studied in relation to cancer, Mortalin is overexpressed in a variety of tumors, including breast, pancreatic, lung, and ovarian cancers, and it is associated with multiple processes of carcinogenesis, which include the inactivation of tumor suppressor p53, deregulation of apoptosis, activation of EMT, and induction of cancer cell stemness. [56,57,58,59,60]. GRP78, a resident protein in endoplasmic reticulum (ER), is also overexpressed in multiple cancers, which are basally subject to ER stress. GRP78 serves as a survival factor for cancer cells as it prevents ER-stress related autophagy and apoptosis [11]. In HSP70-overexpressed cancer cells, HSP70 may translocate to plasma membrane or can be extracellularly released, where it mediates antitumor immune responses [61]. Although the function of extracellular HSP70 regarding carcinogenesis is largely unknown, the extracellular form may provide an additional advantage to cancer cells by stimulating the immune system to remove the unwanted cells from circulation [62]. Intriguingly, extracellular HSP70 forms the activation complex with various co-chaperones, including HSP90α, Hop, and HSP40, which together promote the migration and invasion of the breast cancer cells via the enhanced activity of MMP2 [63]. HSP70 can also be localized on the endolysosomal membrane of cancer cells and serves to resist lysosomal cathepsine-induced cell death [64].

### 2.5. Role of HSP90 in Cancer Development

HSP90 is the most studied HSP family for its numerous implications in cancer development. Like HSP27 and HSP70, HSP90 family inhibits cellular apoptosis and plays important roles in the folding, stabilization, activation, and proteolytic degradation in multiple cancers [65]. HSP90 family consists of five members that are encoded by the HSPC1-5 genes which modulate tumor growth, adhesion, invasion, metastasis, angiogenesis, and apoptosis [51]. Many studies have reported that HSP90 is often overexpressed and associated with poor prognosis in multiple tumors, including cholangiocarcinoma, lung, gastric, and breast cancers and glioblastoma [8,66,67,68,69]. The increased expression of HSP90 promotes carcinogenesis through regulation of correct folding, stability, and function of numerous oncogenic proteins. HSP90 exerts the structural stabilization of the mutated form of p53, which suppresses the growth arrest and apoptosis in response to cell stressors such as DNA damage [70]. The increased expression of HSP90 promotes the activation of oncogenic protein kinases, which are JAK2/STAT3, PI3K/AKT, and MAPK, and facilitates the cancer cell progression [71]. It has also been demonstrated that HSP90 physically interacts with the promoter of human telomerase reverse transcriptase (hTERT), whose expression is frequently enhanced during cellular immortalization, and is responsible for the enhanced telomerase activity in cancer cells [72]. In addition, HSP90 activates HIF-1α and NF-kB, which together enhance the oncogenic events such as cancer cell EMT, invasion, and motility that together confer metastasis of cancer [73]. Furthermore, HSP90 interacts with and inhibits the degradation of 3-hydroxy-3-methylglutaryl-CoA reductase (HMGCR), the rate-limiting enzyme of mevalonate pathway that is essential for cancer progression [74]. Since HSP90 serves to promote the transcription and expression of vascular endothelial growth factor receptors (VEGFRs), the major receptors involved in endothelial cell-dependent tumor angiogenesis, HSP90 overexpression leads to the enhanced proliferation, migration, invasion, and tube cell-dependent tumor angiogenesis in vitro and in vivo [75]. In breast cancer, increased levels of HSP90 are often detected, and HSP90 functions to stabilize the heightened activation of estrogen receptor (ErbB)-dependent PI3K/AKT and ERK signaling pathways. This effect reverses the anti-cancer effects of the hormonal drug Fulvestrant that aims to deactivate the pathways [76]. In addition, HSP90 plays a critical role in prostate cancer formation as it serves to stabilize androgen receptor in a conformation with better affinity for androgen. Therefore, heightened levels of HSP90 are detected in prostate cancer cells [77]. 

### 2.6. Role of HSF1 in Cancer Development

Heat shock transcription factor 1 (HSF1) is a master regulator of all heat shock responses and overexpressed in various cancers [78]. In fact, the increased expression and activity of HSF1 are observed in a variety of cancers, such as prostate and breast cancers, in advanced stages, suggesting HSF1 as an important regulator of tumor progression and metastasis [79,80,81,82,83]. As the upstream modulator of various pathophysiological proteins such as HSP70, HSP90, MIF, Bcl2, and Bax, HSF1 assists in multiple parts of malignancy, including proliferation, migration, invasion, and inhibition of apoptosis [84,85]. Furthermore, HSF1 functions to allow cancer cell formation and progression by inducing genomic instability and stabilizing mitotic spindle organization through regulation of Cyclin D, p21, and p27 during mitosis [86,87,88]. It has recently been reported that the phosphorylation of HSF1 by PIM2, which interrupts the binding of the E3 ligase to HSF1, enhances the stability of HSF1, and promotes HSF1 binding to PD-L1 promoter. The resulting overexpression of PD-L1 enhances the breast cancer growth and tumorigenesis in vitro and in vivo [89]. In addition, Family with sequence similarity member C (FAM3C) is a commonly known interleukin-like EMT inducer, and it has been demonstrated that the upregulation of HSF1 is the underlying mechanism of FAM3C-mediated tumorigenesis [90]. In gastric cancer, the overexpression of HSF1 has been observed in the patient samples, suggesting HSF1 to be the poor prognosis factor [91,92]. Regarding cancer cell defense mechanism, it has been shown that HSF1 forms a positive feedback loop with pyruvate dehydrogenase 3 (PDK3) to drive chemoresistance of cancers [93]. In hepatocellular carcinoma, HSF1 reduces the anti-cancer effects of epirubicin and increases the cell viability by promoting protective autophagy through upregulation of ATG4B expression [94].

## 3. Role of Heat Shock Proteins in Chemotherapy Resistance

As HSPs play protective roles in response to stress conditions that induce protein denaturation and apoptosis, HSPs also play important roles in inducing cancer cell resistance to various drugs used in anti-cancer chemotherapy [35]. In this review, their roles in chemotherapy resistance in different cancer types are discussed and summarized in Table 1.

HSP27 is associated with chemoresistance and poor prognosis in multiple cancers, including gastric, liver, prostate, lung, and colorectal cancers [16]. HSP27 enhances multidrug-resistance in squamous cell carcinoma of tongue (SCCT) through hyperactivation of NF-κB. [95]. In ovarian cancer, the increased expression of HSP27 induces cellular resistance against cisplatin therapy by inhibiting p21 transfer from the nucleus [96]. In laryngeal cancer cells, the overexpression of HSP27 exerts cellular resistance against various cytotoxic agents, such as cisplatin and staurosporin, by inducing cell cycle arrest and remodeling actin polymerization involved in drug uptake [97]. In lung cancer, HSP27 promotes TGF-β-induced cisplatin resistance through regulation of SMAD3 [99]. In particular, lung cancer stem cells showed decreased apoptotic response to treatment with superoxide, cisplatin, and gemcitabine when HSP27 was hyperactivated [100].

Although the exact mechanisms are still not known, the cellular modulation of HSP40 and HSP60 has been shown to be implicated in various drug resistance cancer cells. HSP40 hypo-expression was observed in the ovarian cancer lesions that were resistant to the chemotherapeutic agents, including paclitaxel, topotecan, and cisplatin [101]. In another study, inhibition of DnaJB8 restored the drug sensitivity to docetaxel in renal cell carcinoma [102]. The epigenetic inactivation of DnaJD1, observed in malignant pediatric brain tumors, further indicated for its potential role in disease pathogenesis and chemotherapeutic resistance [103]. HSP60 is highly expressed in oxaliplatin- and cisplatin-resistant ovarian and bladder cancer cells compared to the nonresistant cancer cells [104]. Notably, inhibition of HSP60 enhanced the drug sensitivity of 5-FU-resistant colorectal cancer cells, suggesting that HSP60 may play an important role in 5-FU resistance [104]. However, more investigations are needed to investigate for the role of HSP40 and HSP60 in exerting cellular resistance against chemotherapeutic agents.

As a major HSP that promotes cellular evasion of apoptosis, the association of HSP70 with anti-cancer drug resistance of various cancer cells has been extensively studied. HSP70 is highly expressed in cisplatin-resistant cancer cells, including ovarian cancer, lung cancer, and osteosarcoma. The underlying mechanisms of HSP70-mediated chemoresistance include disruption of mitochondrial apoptotic cascade and maintenance of cell cycle progression [105,106,107,108]. Mortalin (HSP70) overexpression is associated with cisplatin resistance of ovarian cancer cells [109]. In addition, the elevated expression of GRP78 (HSP70) is implicated in 5-FU resistance of colorectal and ovarian cancer cells through modulation of PI3K/AKT/mTOR and c-Src/LSF/TS signaling axes, respectively. [108,110].

Various drug-resistant cancer cell lines have increased expression of HSP90 and concomitantly increased activations of pro-survival signaling pathways and cell cycle progression. The expression of HSP90 is induced by the commonly used chemotherapeutic agents, which are doxorubicin, cisplatin, and methotrexate [113]. A mechanism study indicated that the increased HSP90 expression along with its client proteins, EGFR, IGF-1R, and Src, promotes autophagy in cancer cells and confers drug resistance [114]. In addition, HSP90 regulates the expression of various drug resistant genes, including LRP, GST-π, p53, bcl-2, survivin, ERCC1, XRCC1, BRCA1, and BRCA2 [115].

## 4. Role of Heat Shock Proteins in Radiotherapy Resistance

Radiotherapy along with surgery and chemotherapy has been one of the most commonly used cancer therapies for almost a century. RT combined with standard chemotherapeutic drugs focuses on sensitizing cancer cells to ionizing radiation (IR), which damages DNA of cancerous tissue and induces cell death [118]. DNA Damage Response Pathway (DDR) occurs shortly after the creation of single or double strand breaks that result from IR. An increasing body of work proposes that DDR proteins, such as ATR, FANCA, RAD51, and BRCA2, are kept active by HSP70 and HSP90. Also, HSP70 and HSP90 are directly related to cell cycle regulators, including CHK1, WEE1, CDK1, and CDK4 [119,120]. Therefore, the adjuvant therapies that increase tumor sensitivity to IR through modulation of the proteins that affect the expression and/or activation of HSP70 and HSP90 proteins, have been suggested [121].

The elevated expression of HSP70 has been observed in various cancers that exhibit resistance to radiotherapy, including lung cancer, breast cancer, tongue cancer, and gingiva cancer [122]. HSP70 is induced by HSF1, which is the main factor involved in the transcription of HSP70. Under radiotherapy treatment, HSF1 is transported into the nucleus and binds to the heat shock element in the promoter region of HSP70. This inducible overexpression of HSP70 in human cancers is associated with poor prognosis and resistance against radiotherapy [123]. HSF1 and HSP70 are already overexpressed in tumor cells under physiological conditions, and therefore, RT exacerbates the therapy resistance in the vicious manner [124]. To overcome the tolerance, several strategies have been proposed to limit the expression of HSP70 under radiotherapy. Du et al. discovered that siRNA-mediated inhibition of HSP70 effectively enhances radiotherapy efficacy in endometrial cancer cells [125]. A recent study discovered that the constitutive overexpression of Redd1, which positively regulates the expression of HSP70 through AKT phosphorylation, is observed in IR-resistant lung cancer cells compared to that in normal lung tissues, suggesting Redd1 as a novel adjuvant target [126]. Meanwhile, the extracellular form of HSP70 tethered to the cellular membrane can be induced by radiotherapy, suggesting that the immunotherapy following after radiotherapy may be an effective combinatorial strategy [127]. Inhibition of HSP70 by peptide aptamer A17 exerts the radiosensitizing effect on breast and lung cancers as the co-administration of aptamer A17 and Hsp90 inhibitor NVP-AUY922 significantly enhance DNA double-strand breaks and cell cycle arrest in cancer cells [128].

HSP90-mediated signaling was identified as the main pathway associated with cancer cell resistance to radiotherapy [129,130,131]. Accordingly, targeting HSP90 using numerous candidate inhibitors has been regarded as an attractive strategy to sensitize various cancers to radiotherapy. HSP90 inhibitor NW457, synergized with IR therapy, induces CRC cell apoptosis by inhibiting DDR and abrogating clonogenic survival in vitro and in vivo [132]. The use of HSP90 inhibitor AUY922 with platin-based radiation has shown efficacy in synergistic killing of mutant head and neck squamous cell carcinoma (HNSCC) cells through chromosomal fragmentation [119]. HSP90 inhibitor, Ganetespib, has been identified as a radiosensitizer that works via modulation of HIF-1α, STAT3, and AKT-driven pathways in pancreatic ductal adenocarcinoma [133]. Also, Ganetespib significantly limits the cancer cell survival by inducing G2-M arrest and disrupting DDR during irradiation [134]. PU-H71, Hsp90 inhibitor, exhibits therapeutic efficacy in inhibition of cell survival and accumulation of DNA damage by suppressing RAD51 and Ku70 expression; it is currently evaluated in clinical trials [135]. PU-H71 sensitizes tumor cells to Carbon-ion radiotherapy (CIRT) through interruption of homologous recombination and non-homologous end-joining machineries. Similarly, TAS-116 suppresses cancer cell survival under radiotherapy by interrupting the double-strand break repair systems [136]. In lung cancer, the combined approach that involves HSF-1 knockdown and HSP90 inhibitor NVP-AUY922 inhibits the expression and activation of HSP90 client protein Akt and impairs Rad51-mediated homologous recombination [137] (Table 2).

## 5. Role of Heat Shock Proteins as Immunomodulants

As HSPs are overexpressed in cancer cells under physiological conditions, immunotherapy that targets cancer-derived HSPs has recently been suggested as a novel strategy. The use of HSPs in enhancing the effects of immunotherapy is summarized in Table 3. HSP27 and HSP90 are highly expressed in myeloma cells and have been identified to be naturally expressed in the context of major histocompatibility complex class I (MHC I) molecules. The treatment of cytotoxic T lymphocytes engineered to target HSP27- and HSP90-specific peptides effectively decreased the tumor growth in a myeloma xenograft mouse model, suggesting the HSPs as tumor associated antigens (TAA) for myeloma immunotherapy [138]. Similarly, DnaJB8, which is HSP40 subfamily, is highly expressed in cancer stem-like cell/cancer-initiating cell (CSC/CIC) isolated from colorectal cancer compared with non-CSC/CIC. As CSC/CIC have been thought to be essential for tumor maintenance, recurrence, and distant metastasis, the engineering and administration of DnaJB8-specific cytotoxic T lymphocytes exhibited a significant anti-cancer activity in vivo [139].

HSP70 has been found to be secreted from cancer cells, and, unlike the intracellular form, extracellular HSP70 proteins prompt cytotoxic lymphocytes to target and kill the cancer. An unusual cell surface localization of HSP70 has been demonstrated by a variety of solid tumors, including lung, breast, colorectal, and pancreatic cancers. The membrane HSP70 phenotype has been associated with tumor malignancy, characterized by increased invasion, metastasis, and resistance to cell death. Intriguingly, natural killer (NK) cells, but not T cells, have been found to kill membrane HSP70 positive tumor cells [147]. Ex vivo activation of NK cells with a naturally occurring HSP70 peptide and IL-2 (TKD-IL-2) enhanced the anti-cancer ability of NK cells against lung cancer and glioblastoma in preclinical tumor models [144]. Many vaccines, therefore, have been developed on the basis of ability of HSP70 to efficiently reconfigure antigen presenting system. Guzhova and Margulis discovered that the continuous intratumoral administration of HSP70 inhibits the tumor progression and thus delays the tumor growth. The treatment has been shown to increase the survival of cancer-bearing rats by enhancing the infiltration and activation of NK cells and T lymphocytes through the upregulation of IFNγ production [143]. Recently, the fusion of dendritic cells and whole tumor cells to generate DC-tumor fusion cells (DC-tumor FCs) has been developed to effectively deliver TAAs to dendritic cells as cancer vaccines. The cell fusion method facilitates dendritic cells to be exposed to the broad array of TAAs originally expressed by whole tumor cells, thereby stimulating antitumor immunity through simultaneous activation of both CD4+ and CD8+ T cells. Whereas tumor-derived HSP70-peptide complexes have shown the limited immunogenicity in large-scale phase III trials, HSP70-peptide complexes derived from DC-tumor FCs have shown the enhanced immunogenicity and induced much more powerful immune responses against breast cancer [145].

Other than suppressing the expression and activation of oncogenic proteins, HSP90 inhibition has recently been found to positively modulate the effect of cancer immunotherapy [148]. HSP90 inhibition using Ganetespib has been shown to increase the expression of interferon response genes, such as IFIT1, IFIT2, and IFIT3, which promote killing of melanoma cells by T cells. The combination of Ganetespib and anti-PD1 or anti-CTLA4 conferred a better anti-tumor response in mouse model, compared to either treatment alone [140]. In addition, Haggerty et al. investigated that twelve different HSP90 inhibitors increase the expression of melanocyte differentiation antigens, Melan-A/MART-1, gp100, and TRP1, as well as MHC Class I. This finding suggests that HSP90 inhibition facilitates recognition of tumor cells by T cells by increasing the expression of intracellular antigen pool available for processing and presentation by MHC Class I, along with the increased expression of MHC Class I itself [141]. Furthermore, HSP90 inhibitor 17-MAG has been indicated as an immune adjuvant in the context of vaccines targeting HSP90 client protein EphA2, reconditioning the tumor microenvironment to enhance patient immune responses. The co-treatment of 17-MAG and antibody against HSP90 client protein EphA2 effectively reduces immune suppressor cell populations, such as myeloid-derived suppressor cell and regulatory T cells, while recruiting Type-1 T effector cells through chemokines such as CXCL10 and enhancing the recognition of tumor cells by CD8+ T cells [142]. GRP94 is an ER resident HSP90 paralog that plays an essential role in the folding of Toll-like receptors (TLRs) and integrins, suggesting GRP94 as a specialized immune chaperone that controls receptors for immune function [149,150]. As a master immune chaperone, GRP94 is associated with early B- and T-cell development and regulates the innate immune response of macrophage and regulatory T cells [151,152]. In addition, GRP94 regulates the activity of tumor-associated macrophages through folding of TLRs and integrins [153]. Genetic deletion of GRP94 from macrophages decreased the inflammation-associated colon tumorigenesis by suppressing the production of pro-inflammatory cytokines such as IL-17 and IL-23 [146].

## 6. HSP Inhibition as a Potential Strategy to Effectively Cure Cancer

### 6.1. HSP27 Inhibition for Cancer Therapy

Since HSP27 induces therapeutic resistance against radiotherapy and chemotherapy, HSP27 may serve as a potential targeting molecule for cancer therapy [96,100,126,154]. HSP27 inhibitors, such as quercetin and RP101, enhance the anticancer therapeutic effects in various cell lines, including leukemia, glioblastoma, and oral cancer cells [155,156,157,158]. In human leukemia, quercetin in combination with shHSP27 synergistically inhibits cell proliferation and promotes apoptosis by decreasing Bcl2 to Bax ratio. In addition, the combinatorial administration significantly suppresses the infiltration of tumor cells by decreasing the activation of Notch/AKT/mTOR signaling pathway and down-regulating the expression of angiogenesis-associated proteins, HIF1α and VEGF [155]. Moreover, treatment of quercetin suppresses the expression of autophagy-associated protein Atg7, thereby promoting cancer cell death through autophagy blockade, when co-administrated with cytotoxic agent t-AUCB [156]. Another HSP27 inhibitor, RP101, is an antiviral nucleoside, also known as bromovinyldeoxyuridine, and binds through π-stacking with Phe 29 and Phe 33 of HSP27. This agent interrupts HSP27 interaction with client proteins and functions to sensitize tumor cells to chemotherapy [159]. In a stage II clinical study, RP101 enhanced the survival of pancreatic cancer patients in combination with chemotherapeutic agent gemcitabine (NCT00550004) [159]. In addition, TDP, a natural HSP27 inhibitor extracted from Chinese traditional medicinal herb Garcinia oblongifolia, suppresses levels of HSP27 and induces cancer cell death [160,161]. Another approach to target HSP27 is to use the peptide aptamer (PA) that limits the structural flexibility of HSP27 and impairs HSP27- dependent anti-apoptotic and cyto-protective activities [162]. Two peptide aptamers, PA11 and PA50, have been shown to limit the structural changes of HSP27 and decrease its anti-apoptotic and tumorigenic activities [163]. While the use of HSP27 is implicated in antisense therapy, OGX-427 (apatorsen) is an antisense oligonucleotide that decreases the expression of HSP27 and is currently progressed in Phase II clinical trials. Treatment with OGX-427 has incurred the decreased metastatic ability of prostate cancer cells [164] (Table 4).

### 6.2. HSP40 Inhibition for Cancer Therapy

Although the exact underlying mechanism needs further investigations, regulation of HSP40 family proteins have been implicated in various chemotherapeutic agents and shown to enhance the anti-cancer activity. KNK437, a benzylidene lactam compound and a pan-HSP inhibitor, inhibits the expression of HSPs, which are HSP27, HSP40, HSP 72, and HSP110 [165]. Treatment of colorectal cancer cells with KNK437 inhibits the expression of DnaJA1 and the cell cycle progression through destabilization of CDC45 [32]. As DnaJB1 positively regulates the epidermal growth factor receptors (EGFR) signaling, knockdown of DnaJB1 promotes the sensitivity of tumor cells to anti-cancer effects of the EGFR inhibitor gefitinib in human lung epithelial adenocarcinoma cells [31]. In non-small cell lung cancer, BMS-690514, an inhibitor of human EGFR and vascular EGFR, induces G1 cell cycle arrest and stimulates caspase-dependent apoptosis through downregulation of HSP40 and other HSPs [166]. Inhibition of DnaJB8 also induces the sensitivity of kidney cancer cells to decetaxel [102]. Phenoxy-N-arylacetamides is known to significantly inhibit the expression of HSP40, but it is still in the early stages of development to be confirmed as a therapeutic tool [167]. In colorectal cancer cells, knockdown of DnaJB6 suppresses the cancer metastasis by decreasing IQ-domain GTpase-activating protein 1 and phosphorylated ERK. Silencing DnaJB6 with siRNA exerts the genotoxic stress/p53-induced apoptosis in human neuroblastoma, osteosarcoma, and lung cancer [29] (Table 5).

### 6.3. HSP60 Inhibition for Cancer Therapy

HSP60 has been implicated in anti-cancer drug activities and drug resistance. Loss of mitochondrial HSP60 pool has been implicated in geldanamycin (GA)-induced killing of osteosarcoma cells, suggesting that targeting HSP60 may be the underlying mechanism of GA-mediated cytotoxicity [171]. Myrtucommulone (MC), a nonprenylated acylphloroglucinol present in the leaves of myrtle, directly interacts with HSP60 to induce mitochondrial related apoptosis [168]. Sinularin, a bioactive compound derived from coral *Sinularia flexibilis*, exhibits the anti-cancer effects through upregulation of pro-apoptotic caspase system by decreasing the expression of HSP60 in A2058 melanoma cells [169,170]. In regard to therapy resistance, the increased expression of HSP60 enhances the extent of cancer cell resistance to platinum analogs in human ovarian and bladder carcinoma cells, and, therefore, inhibition of HSP60 in 5-FU resistant SW480 CRC cells induces the attenuation of drug resistance [172]. As an interesting side note, a proteasome inhibitor Bortezomib assists in the anti-cancer treatment via upregulation of HSP60 and HSP90 on the surface of cancer cells, facilitating cancer cell recognition by dendritic cells [170] (Table 5).

### 6.4. HSP70 Inhibition for Cancer Therapy

HSP70 is highly expressed in various cancers and associated with tumorigenesis and drug resistance [11]. Over the last decade, various studies have been conducted to advance the development of HSP70 inhibitors for cancer therapy. Fisetin, a dietary flavonoid, is known to induce cell apoptosis in HCT-116 colon cancer cells by inhibiting HSF1 from binding to the promoter region of HSP70 and BAG3. Since HSP70/BAG complexes protect cancer cells from apoptosis through stabilization of anti-apoptotic Bcl-2 family member proteins, the downregulation of HSP70/BAG3 significantly reduces the levels of BCL-2, BCL-XL, and myeloid cell leukemia 1 (MCL-1) proteins in human HCT-116 colon cancer cells [173]. Pifithrin-μ (PES), also known as 2-phenylethynesulfonamide, is a HSP70 inhibitor that exerts anti-cancer effects in various cancer types, such as non-small cell lung cancer (NSCLC), through G0/G1 phase cell cycle arrest and promotion of the death receptors 4 and 5 expression [174]. However, it has recently investigated that PES may increase intracellular ROS levels and promote the metastatic behavior of surviving cells [175]. Cantharidin (CTD) is a terpenoid derivative isolated from blister beetles and inhibits the expression of HSP70 by blocking HSF1 from binding to HSP70 promoter [176]. Administration of thermal-sensitive liposomes encapsulating CTD induces cell apoptosis by blocking heat shock response and the subsequent expression of HSP70 and BAG3 in human cervical cancer [177]. In addition, Apoptozole (AZ) is a HSP70 inhibitor that promotes cancer cell apoptosis through lysosomal membrane permeabilization. AZ-mediated impairment of lysosomal function also inhibits the protective autophagy and promotes cell apoptosis in multiple cancer cell lines [178]. 

Since HSP70 promotes the resistance of cancer cells to chemotherapy, the strategies that chemo-sensitize cancer cells through HSP70 inhibition have been explored. HSP70 knockdown is suggested as an adjuvant strategy to enhance the apoptotic effect of cisplatin in cervical cancer [179]. Furthermore, as HSP72 (HSP70) stabilizes stromal cell-derived factor 2 (SCF2) that protects human gastric cancer cells against oxaliplatin, inhibition of HSP72 (HSP70) to debilitate the protective function of SCF2 was suggested [180]. Moreover, overexpression of HSP72 (HSP70) induced by bortezomib was found to limit the anti-cancer effects of bortezomib, and combination therapy with HSF1 inhibitor was effective in enhancing bortezomib-mediated cancer cell death [181]. To overcome the inducible overexpression of HSP70, HS-72, an allosteric inhibitor selective for the inducible form of HSP72, has been posed as an effective agent to limit the expression of HSP70 after anti-cancer treatments [182]. In addition, abnormal phosphorylation of HSC70 (HSP70) has been found to inhibit transportation of methotrexate anti-cancer agents into tumor cells, suggesting de-phosphorylation of HSC70 as a viable strategy to enhance the sensitivity to methotrexate [183]. Tissue microarray analyses suggested Mortalin (HSP70)-positive tumor cells exhibit the increased resistance against cisplatin, and silencing Mortalin with shRNA enhanced the drug sensitivity to cisplatin and reduced the tumor cell growth in ovarian cancer [109]. In addition, MKT-077, a cationic rhodacyanine dye that inhibits Mortalin (HSP70), suppresses the cell viability of ovarian cancer and blocks the EMT progression through inhibition of Wnt/β-Catenin signaling [184]. Embelin, a natural quinone derived from the fruits of *Embelia ribes*, exerts the anti-cancer effect by limiting the inhibitory function of Mortalin (HSP70) on p53. In breast cancer, the treatment of embelin, therefore, reduced cancer cell growth and metastatic ability by activation of p53 [185]. Veratridine (VTD) has shown efficacy in selectively suppressing the expression of Mortalin (HSP70) by increasing the expression of a ubiquitin-like protein called UBXN2A that degrades Mortalin (HSP70) [186]. In addition, knockdown of GRP78 (HSP70) prompts 5-FU-induced apoptosis through deterioration of ER stress [108]. Isoliquirtigenin, a chalcone-type flavonoid derived from licorice root, directly targets GRP78 (HSP70) and suppresses cancer cell colony formation through inhibition of GRP78 (HSP70)-mediated β-catenin/ABCG2 signaling in breast cancer stem cells [187,188] (Table 6).

### 6.5. HSP90 Inhibition for Cancer Therapy

HSP90 has been the effective anti-cancer therapeutic target for its extensive associations with tumor initiation, development, metastasis, and resistance to anti-cancer drugs. A variety of HSP90 inhibitors have been evaluated in clinical trials for cancer therapy. Some of the clinical trials are completed (NCT01294202, NCT01685268, NCT00878423, NCT01246102), and others are recruiting more patients. Ganetespib, a second-generation synthetic HSP90 inhibitor that has exhibited promising antitumor effects with safety profiles, is being developed to treat metastasis-prone and drug-resistant thyroid, ovarian, breast, and non-small cell lung cancers, currently undergoing clinical trials [189,190,191,192]. Ganetespib effectively suppresses cancer progression by inducing G2/M cell cycle arrest through inhibition of RAS/RAF/ERK and PI3K/AKT/mTOR pathways and promoting caspase-3-mediated apoptosis [189,193]. Another HSP90 inhibitor, NVP-AUY922, is a novel resorcinylic isoxazole amide that decreases cancer cell viability through suppression of cancer-derived survivin protein levels, which functionally interfere with the cell modalities for growth inhibition and cell apoptosis [194]. As an adjuvant therapy, co-administration of HSP90 inhibitor, NVP-AUY922, with ABT-737, BCL-2 inhibitor, enhances the anti-cancer effect of ABT-737 by targeting MCL-1 protein that grants the cancer resistance to ABT-737 in small cell lung cancer [195]. Likewise, NVP-AUY922 inhibits cell growth in HER2-positive and trastuzumab-resistant breast cancer cells [196]. PU-H71 is a next-generation HSP90 inhibitor that exhibits the anti-cancer activity in multiple tumors. In chronic lymphocyte leukemia (CLL), PU-H71 decreases B-cell receptor (RCR) kinases and induces CLL cell apoptosis under cytoprotective conditions [197]. In addition, PU-H71 inhibits PI3K/mTOR pathway in Burkitt lymphoma through MYC dysregulation [198]. Panaxynol is a natural compound that elicits anti-cancer activity through inhibition of the HSP90 expression and induction of apoptosis [199]. Geldanamycin, extracted from *Streptomyces hygroscopicus*, elicits the anti-cancer activity through blockage of ATP-binding site of HSP90 and inhibition of its function [200]. Geldanamycin also restores cancer cell sensitivity to paclitaxel by inactivation of p38/H2AX axis in paclitaxel-resistant ovarian cancer cells [201]. The natural product, gambogic acid (GBA), binds selectively to the middle domain of HSP90β [202]. In pancreatic cancer, the administration of GBA suppresses cell proliferation by inducing cell cycle arrest and apoptosis. In addition, GBA attenuates the evolution of cancer cell resistance to gemcitabine by inhibiting ERK/E2F1/RRM2 signaling pathway [203]. TAS-116, a selective cytosolic HSP90α and β inhibitor that does not inhibit HSP90 paralogs such as GRP94, has been shown to exert antitumor activities by depleting levels of several HSP90 client proteins [204]. In the clinical phase I study, the administration of TAS-116 garnered an acceptable safety profile for the patients with non-small cell lung cancer and gastrointestinal stromal tumor [205]. In prostate cancer, the C-terminal HSP90 inhibitor KU675 exerts cytotoxic effects by inhibiting the formation of HSP90 complexes and promoting degradation of HSP90 client proteins [206]. Other C-terminal HSP90 inhibitors, SM253 and SM258, have also been shown to inhibit cell proliferation and induce apoptosis in several prostate cancer cell lines. Unlike N-terminal inhibitors, such as AUY922 and 17-AAG, these C-terminal inhibitors do not increase the expression of HSP27, HSP40, and HSP70, suggesting their efficacy in anti-cancer function [207]. In triple-negative breast cancer, the C-terminal HSP90 inhibitor L80 effectively reduces the cell proliferation, breast cancer stemness, tumor growth, and metastasis by inhibiting AKT/MEK/ERK/JAK2/STAT3 signaling, while not affecting normal cells [208]. GRP94-selective inhibitor 30 has been developed from the structural modification of the first generation cis-amide bioisostere imidazole to improve its affinity for GRP94 [209]. Since GRP94 is responsible for the maturation and trafficking of proteins involved in cell signaling and motility, the treatment of GRP94-selective inhibitor 30 has shown potent anti-cancer activity in aggressive and metastatic cancers [209] (Table 7).

### 6.6. HSF1 Inhibition for Cancer Therapy

Studies have shown that HSF1 plays an important role in cancer cell tumorigenesis, apoptosis, and proliferation and multi-drug resistant in malignant tumors [213,214]. Targeting HSF1-mediated signaling axes may pose an effective strategy to treat cancer. The effects of suppressing the function of HSF1 in cancer types such as breast cancer, colorectal cancer, and leukemia have been examined [215]. The inhibition of HSF1 led to the decreased breast cancer formation and lung metastasis in a mouse model [216]. In colorectal cancer, the knockdown of HSF1 inhibited mTOR activation and glutamine metabolism while attenuating the cancer cell growth in vitro [215]. It has also been demonstrated that the inhibition of HSF1 reduces the invasion, migration, and EMT of pancreatic cancer cells by suppressing activation of AMPK. In addition, HSF1 inhibition decreased tumor growth and increased the overall survival of tumor-bearing mice [217]. In breast cancer, since the activation of AKT and the subsequent activation of HSF1 by p-AKT shortens the metastatic intervals of tumor cells, inhibition of the AKT/HSF1 signaling axis resulted in the reduced number of metastatic breast cancer cells and cancer stem cells [80]. Currently, several HSF1 inhibitors have been identified for the treatment of cancer, although the therapeutic effects are still being evaluated. An HSF1 inhibitor, 2,4-Bis(4- hydroxybenzyl)phenol, extracted from rhizomes of *Gastrodia elata*, induces the degradation of HSF1 through the impairment of HSF1 protein stability. The decreased level of HSF1 is accompanied by attenuated levels of HSPs, such as HSP27 and HSP70. In lung cancer, the HSF1 inhibitor induced cancer cell growth arrest and apoptosis and helps to overcome cancer cell resistance to conventional anti-cancer drugs, including paclitaxel and cisplatin [218]. From the phenotypic screen targeting HSF1 heat shock pathway with a chemically diversified library of over 100,000 compounds, PW3405 has been identified to inhibit phosphorylation and activity of HSF1 and suggested to inhibit cancer cell viability in a broad range of tumors in vitro [219]. A synthetic HSF1 inhibitor, I_HSF_115, inhibits the transcription activity of HSF1 by interfering with the assembly of the transcription complexes. I_HSF_115 exhibits high cytotoxicity for multiple cancer and myeloma cell lines, repressing a large majority of heat-induced genes [220] (Table 8).

## 7. Discussion

HSPs are molecular chaperones that are induced under cellular stress and often overexpressed in many cancers. The major HSPs, including HSP27, HSP40, HSP60, HSP70, HSP90, are associated with several key oncogenic drivers for tumorigenesis. In the clinical context, the abnormal levels of HSPs may indicate poor prognostic outcomes. In addition, the major HSPs that serve as diagnostic biomarkers of cancer have been discussed in relation to their roles in tumorigenesis, evasion of apoptosis, cell invasion, and metastasis. The functions of HSPs are diverse and elicit the hallmarks of cancer, as their roles are essential in initiation, development, and recurrence of cancer. At the same time, their differentiated roles share a similar level of importance in paving the way towards understanding cancer and developing effective treatments.

As noted above, HSPs play important roles in cancer development and resistance to chemo-, radio-, and immunotherapy treatments. Accordingly, targeting HSPs has been discussed as a necessary standalone or adjuvant therapy to overcome the limitations of current anti-cancer treatments. In fact, several anti-cancer drugs that inhibit HSP client proteins have already been approved by the FDA. For instance, HSP70 client protein inhibitor sorafenib has been approved to treat renal carcinoma, hepatocellular carcinoma, and thyroid carcinoma (25858032). Moreover, candidate anti-cancer drugs that modulate the function of HSPs have been investigated in numerous preclinical lab settings and clinical trials. To date, however, there are no FDA-approved HSP inhibitors available. Regardless, future HSP-associated drugs may directly target HSPs, regulating the downstream effectors of HSPs and enhancing the anti-cancer effects of various therapies met with their own limitations. Furthermore, understanding the role of HSPs in immunotherapy settings poses a significant opportunity. Several studies have demonstrated that modulation of HSPs may stimulate the innate, cell-mediated, and humoral immune responses. The use of HSPs as target antigens or activators of the immune responses is a novel strategy to regulate immunostimulatory. To meet these ends, understanding the functions and molecular mechanisms of HSPs is essential and requires continuous efforts.

## Figures and Tables

**Table 1:** Role of heat shock proteins in chemotherapy resistance.

Name	Cancer Type	Findings	Reference
HSP27	Squamous cell carcinoma of tongue	Induction of multidrug-resistance by hyperactivating NF-κB signal and suppressing mitochondrial caspase signal Reduction of chemoresistance via HSP27 knockdown and its antibody treatment	[95]
	Ovarian cancer	Induction of cisplatin resistance by inhibiting p21 via activation of AKT pathway	[96]
	Laryngeal cancer cell	Induction of chemoresistance to cisplatin and staurosporin by delaying cell growth and remodeling actin polymerization	[97]
	Pancreatic cancer	Induction of chemoresistance to gemcitabine by activation of Snail and ERCC1 and decrease of E-cadherin	[98]
	Lung cancer	Blockage of TGF-β-mediated cisplatin resistance and decrease of cell viability, and increase of cell apoptosis by knockdown of HSP27	[99]
	Lung cancer stem cells	Decrease of apoptotic response treated with superoxide, cisplatin, gemcitabine by activating HSP27/p38/MAPKAK2 and inactivating apoptosis signal	[100]
HSP40	Ovarian cancer	Induction of multidrug resistance, such as paclitaxel, topotecan, and cisplatin	[101]
	Renal cell carcinoma	Induction of chemoresistance to docetaxel by DnaJB8	[102]
	Malignant pediatric brain tumor	Inactivation of DnaJD1, potential role in pathogenesis and chemotherapeutic resistance	[103]
HSP60	Ovarian and bladder cancer	Induction of chemoresistance to oxaliplatin and cisplatin	[104]
	Colorectal cancer	Enhancement of drug sensitivity to 5-FU by inhibiting HSP60	[104]
HSP70	Lung and ovarian cancer, osteosarcoma	Induction of chemoresistance to cisplatin and 5-FU	[105,106,107,108]
	Ovarian cancer	Enhancement of drug sensitivity to cisplatin by increasing mitochondrial cytochrome c release via inhibition of Mortalin	[109]
	Colorectal and ovarian cancer	Acquirement of 5-FU resistance via regulation of PI3K/AKT/mTOR and c-Src/LSF/TS signal by GRP78	[108,110]
	Cervical cancer	Induction of apoptosis by regulating mitochondrial related proteins via GRP78 knockdown	[111]
	Osteosarcoma	Decrease of HSP70 expression by miR-223, deactivation of JNK/JUN signal, and enhancement of cisplatin sensitivity	[106]
	Non-small cell lung cancer	Promotion of cellular resistance to EGFR tyrosine inhibitors by enhancing gene mutation and tumor heterogeneity via inhibition of HSP70	[112]
HSP90	Osteosarcoma	Induction of chemoresistance by inducing autophagy via PI3K/AKT/mTOR pathway and inhibiting of apoptosis via JNK/p38 pathway	[113]
	Colon cancer	Acquirement of drug resistance by activating HSP90 client proteins, such as EGFR, IGF-IR, and Src	[114]
	Ovarian cancer	Regulation of various drug resistant genes, such as LRP, GST-π, p53, bcl-2, survivin, ERCC1, XRCC1, BRCA1 and BRCA2	[115]
	Pancreatic cancer	Induction of drug resistance to 5-FU and gemcitabine by regulating AKT and MAPK and enhancing apoptosis via inhibition of HSP90	[116]
	Breast and gastric cancer	AUY-022 (HSP90 inhibitor), increased effects of lapatinib via inhibition of HER2 and AKT pathway	[117]

**Table 2:** Role of heat shock proteins in radiotherapy resistance.

Name	Cancer Type	Findings	Reference
HSP70	Human glioblastoma	Regulation of HSP70 by HSF1, relation of poor prognosis and resistance against radiotherapy	[123]
	Endometrial cancer	Inhibition of HSP70 by siRNA, promotion of radiotherapy efficacy	[125]
	Lung cancer	Regulation of HSP70 and AKT phosphorylation by Redd1, acquirement of radiotherapy resistance	[126]
	Breast and lung cancer	peptide aptamer A17 (HSP70 inhibition), NVP-AUY922 (HSP90 inhibitor), radiosensitization by increasing DNA double strand breaks and cell cycle arrest	[128]
HSP90	Human gallbladder cancer	NW457 (HSP90 inhibitor), induction of apoptosis by suppressing DDR and survival under IR therapy in CRC	[132]
	Head and neck squamous cell carcinoma	AUY922 (HSP90 inhibitor), sensitization of radiotherapy resistance via chromosomal fragmentation	[119]
	Pancreatic ductal adenocarcinoma	Ganetespib (HSP90 inhibitor), induction of radiosensitization through regulation of HIF-1α, STAT3, and AKT-driven pathways	[133]
	Lung cancer	Ganetespib, inhibition of cancer cell survival via induction of cell cycle arrest and disruption DDR	[134]
	Murine osteosarcoma	PU-H71 (HSP90 inhibitor), inhibition of cell survival and accumulation of DNA damage via decrease of RAD51 and Ku70	[135]
	Lung cancer	NVP-AUY922 (HSP90 inhibitor), inhibition of HSF1, reduction of HSP90 client protein AKT, radiosensitization by impairing the homologous recombination	[137]

**Table 3:** Role of heat shock proteins as immunomodulants.

Name	Cancer Type	Findings	Reference
HSP27 HSP90	Myeloma	Usage of HLA*0201-binding peptides for HSP27 and HSP90, stimulation of peripheral blood cells, production of HSP peptide-specific cytotoxic T lymphocytes (CTLs), induction of cell death, decrease of tumor growth	[138]
HSP90	Human melanoma	Ganetespib—HSP90 inhibitor, enhancement of T-cell induced cell death and anti-CTLA4 and anti-PD1 response, induction of interferon response genes and anti-cancer immune responses of T cells	[140]
	Melanoma	HSP90 inhibitor induced the increase of melanocyte differentiation antigens Melan-A/MART-1, gp-100, TRP-2, and MHC Class I, enhancement of tumor recognition by immune response via increase of MHC class I	[141]
	Melanoma	17-DMAG—HSP90 inhibitor, decrease of EphA2, induction of the recognition of tumor cells by T cells specific antigens	[142]
HSP40 /DnaJB8	Colorectal cancer	Overexpression of DnaJB8 in cancer stem-like cell/cancer-initiating cell, upregulation of stem cell markers and tumorigenesis, production of DnaJB8-specific CTLs by DnaJB8-derived peptide, induction of cell death	[139]
HSP70	Glioblastoma	Administration of HSP70—activation of adaptive immunity, reduction of tumor progression, enhancement of survival, activation of infiltrating NK cells and T lymphocytes, production of IFNγ	[143]
	Non-small cell lung cancer	Activation of NK cells by membrane localized HSP70 peptide (TKD) and IL-2	[144]
	Breast cancer	HSP70 peptide complexes derived from dendritic cell-tumor fusion, enhancement of immunogenicity and immune responses, promotion of T cell activation and CTL responses	[145]
HSP90 /GRP94	Colon Cancer	Genetic deletion of GRP94 from macrophages suppressed the production of IL-17 and IL-23 and decreased inflammation-associated tumorigenesis	[146]

**Table 4:** HSP27 inhibition for cancer therapy.

Name	Cancer Type	Findings	Reference
HSP27	Human leukemia	Quercetin—HSP27 inhibitor, suppression of cell proliferation, induction of apoptosis by decreasing of BCL2/BAX ratio, inhibition of tumor infiltration via inactivation of Notch/AKT/mTOR pathway and HIF1α and VEGF	[155]
	Glioblastoma	Combination of shHSP27 and quercetin—decrease of cancer infiltration and neovascularization related proteins, blockage of cell cycle, induction of autophagy by inhibiting ATG7 expression, acquired chemoresistance	[156]
	Pancreatic cancer	RP101–HSP27 inhibitor, promotion of survival of pancreatic cancer patients combined with gemcitabine, suppression of HSP27 induced resistance	[159] NCT00550004
	Hepatocellular carcinoma	TDP—extraction from Chinese medicinal herb, downregulation of HSP27 expression, induction of apoptosis, decrease of cell growth	[160,161]
	Human cervical cancer and HNSCC	Peptide aptamers—PA11 and PA50, HSP27 targeting, reduction of anti-apoptotic activity of HSP27, decrease of tumorigenesis, inhibition of tumor growth	[163]
	Prostate and bladder cancer	OGX-427—antisense oligonucleotide, suppression of HSP27, decrease of tumor metastasis and circulating cancer cells	[164], NCT01681433

**Table 5:** HSP40 and HSP60 inhibition for cancer therapy.

Name	Cancer Type	Findings	Reference
HSP40	Colorectal cancer	KNK437—pan-HSPs inhibitor, inhibitions of HSP40/DnaJA1, suppression of cell cycle progression by destabilizing CDC45	[32]
	Lung cancer	Knockdown of DnaJB1, enhanced effect of gefitinib (EGFR inhibitor)	[31]
	NSCLC	BMS-690514—hEGFR and VEGFR inhibitor, downregulation of HSP40, promotion of cell cycle arrest and apoptosis	[166]
HSP60	Leukemia	Myrtucommulone—targeting of mitochondrial HSP60, induction of mitochondrial apoptosis	[168]
	Melanoma	Sinularin—HSP60 inhibition, induction of anti-cancer activity, inhibition of cell proliferation and migration, induction of apoptosis	[169]
	Ovarian cancer	Bortezomib—proteasome inhibitor, exhibition of anti-cancer effects, upregulation of HSP60 and HSP90, induction of phagocytosis	[170]

**Table 6:** HSP70 inhibition for cancer therapy.

Targeting HSPs	Cancer Type	Findings	Reference
HSP70	Colon cancer	Fisetin—HSF1 inhibition, reduction of HSP70, induction of apoptosis by inhibiting BCL-2, BCL-XL, and MCL-1	[173]
	Non-small cell lung cancer	Pifithrin-μ—HSP70 inhibitor, inhibition of proliferation via induction of cell cycle arrest, suppression of cell migration, induction of apoptosis	[174]
	Cervical cancer	Cantharidin—HSP70 inhibitor, blockage of HSF1 binding to HSP70 promoter, induction of apoptosis via the inhibition of heat shock response and HSP70 expression	[176,177]
	Cervical, lung colon, and pancreatic cancer	Apoptozole—HSP70 inhibitor, promotion of apoptosis through induction of lysomal membrane permeabilization and impairment of autophagy	[178]
	Ovarian cancer	MKT-077—Mortalin (HSP70) inhibitor, decrease of cell viability, blockage of cell EMT progression, inhibition of Wnt/β-Catenin signaling	[184]
	Breast cancer	HS-72—selective HSP72 inhibitor, reduction of ATP-binding affinity, inhibition of tumor growth, and increase of survival in breast cancer animal model	[182]
	Breast cancer	Embelin—inhibition of Mortalin and p53 interaction, decrease of cell growth and metastasis	[185]
	Colon cancer	Veratridin (VTD)—inhibition of Mortalin through upregulation of UBXN2A	[186]
	Breast and oral cancer stem cells	Isoliquirtigenin—GRP78 inhibitor, inhibition of cell proliferation and colony formation, suppression of β-catenin/ABCG2 signaling Inhibition of cancer stemness, cell proliferation, metastasis and chemoresistance by disrupting ABC transportation	[187,188]

**Table 7:** HSP90 inhibition for cancer therapy.

Targeting HSPs	Cancer Type	Findings	Reference
HSP90	Thyroid, breast, lung and ovarian cancer	Ganetespib—HSP90 inhibitor, inhibition of cell proliferation metastasis, induction of cell cycle arrest, enhancement of apoptosis, decrease of tumor growth	[189,190,191,192]
	Papillary thyroid carcinoma	NVP-AUY922—HSP90 inhibitor, inhibition of cell viability, induction of apoptosis, suppression of survivin	[194]
	Gastric cancer an NSCLC	NVP-AUY922, inhibition of tumor growth, angiogenesis, metastasis	[210,211]
	Small cell lung cancer	Co-administration of HSP90 inhibitor NVP-AUY922 and BCL-2 inhibitor ABT-737—induction of apoptosis, inhibition of ABT-737 drug resistance, downregulation of AKT and ERK	[195], NCT01294202, NCT01685268, NCT00878423, NCT01246102
	Chronic lymphocytic leukemia	PU-H71—HSP90 inhibitor, decrease of B-cell receptor kinase, induction of apoptosis, inhibition of PI3K/mTOR pathway	[197]
	ovarian cancer	Geldanamycin—HSP90 inhibitor, induction of paclitaxel sensitivity, inactivation of p38/H2AX, inhibition of tumor growth, exhibition of structural instability and hepatotoxicity, failure in phase I clinical trials	[201,212]
	Pancreatic cancer	Gambogic acid—selective HSP90β inhibitor, inhibition of cell growth, induction of cell cycle arrest and apoptosis, sensitization of cancer cells to gemcitabine by regulating ERK/E2F1/RRM2 signaling pathway	[203]
	Prostate cancer	KU675—C-terminal HSP90 inhibitor, exhibition of anti-proliferative and cytotoxic activity by suppressing formation of HSP90 complexes and degrading client proteins	[206]
	Prostate cancer	SM253 and SM258—C-termincal HSP90 inhibitor, suppression of cell proliferation, induction of apoptosis, no effect on the expression of HSP27, HSP40, and HSP70	[207]
	Triple-negative breast cancer	L80—C-terminal HSP90 inhibitor, reduction of cell proliferation, cancer stem cell like properties and metastasis by regulating AKT/MEK/ERK/JAK2/STAT3 signaling pathway	[208]
	Lung and gastric cancer	TAS-116—selective HSP90α and β inhibitor, reduction of multiple HSP90 clients, efficient anti-cancer activity	[204,205]
	Breast and prostate cancer	GRP94-selective inhibitor 30—GRP94 inhibitor, potent anti-cancer activity	[209]

**Table 8:** HSP90 inhibition for cancer therapy.

Targeting HSPs	Cancer Type	Findings	Reference
HSF1	Lung cancer	2,4-Bis(4-hydroxybenzyl)phenol—HSF1 inhibitor, inhibition of HSF1 activity, reduction of HSP27 and HSP70 expression, induction of cell growth arrest and apoptosis, mediation of cancer cell drug resistance	[218]
	Prostate, pancreatic cancer and NSCLC	PW3405—HSF1 inhibitor, reduction of HSPs expression, anti-cancer activity, low cytotoxicity to normal cells	[219]
	Lung, ovarian, cervical, breast, prostate cancer and myeloma	I_HSF_115—HSF1 inhibitor, inhibition of HSF1 transcription, repression of heat-induced genes, anti-cancer activity	[220]
